# Cost comparison of laboratory testing vs. empiric treatment for nalaria in Southwestern Nigeria: a prospective study

**DOI:** 10.1186/1475-2875-9-S2-P36

**Published:** 2010-10-20

**Authors:** Ravi Parikh, Isaac Amole, Margaret Tarpley, Daniel Gbadero, Mario Davidson, Sten H Vermund

**Affiliations:** 1 Institute for Global Health, Vanderbilt University School of Medicine, Nashville, TN, USA; 2 Baptist Medical Centre/Bowen University Teaching Hospital, Ogbomoso, Oyo State, Nigeria; 3 Institute for Global Health and Department of Surgery, Vanderbilt University School of Medicine, Nashville, TN, USA; 4 Institute for Global Health and Department of Biostatistics, Vanderbilt University School of Medicine, Nashville, TN, USA; 5 Institute for Global Health and Department of Pediatrics, Vanderbilt University School of Medicine, Nashville, TN, USA

## Background

Presumptive treatment for malaria is common in resource-limited settings, yet controversial given the imprecision of clinical diagnosis. We compared costs of diagnosis and drugs for two strategies: (1) empirical treatment of malaria via clinical diagnosis; and (2) empirical diagnosis followed by treatment only with Giemsa smear confirmation.

## Materials and methods

Patients with a diagnosis of clinical malaria were recruited from a medical center in southwestern Nigeria. The patients received free Giemsa thick (diagnosis) and thin (differentiation) smears, but paid for any antimalarial treatment. Clinical diagnosis was made on clinicians' judgments based on symptoms, including fever, diarrhea, headache, and body-aches. The pediatric regimen was artesunate (6-9 tablets of 3mg/ kg on day one and 1.5mg/kg for the next four days) plus amodiaquine (10mg/kg day 1-2 and 5mg/kg on day three in suspension). Adults were given two treatment options: option one (4.5 50mg artesunate tablets on day one and nine for the next four days, plus three 500mg sulfadoxine/25 mg pyrimethamine tablets) and option two (4.5 50mg artesunate tablets on day one and nine for the next four days plus nine 200mg tablets of amodiaquine at a dose of 10mg/kg day 1-2 and 5mg/kg on day three). We calculated the costs of smears/drugs from standard medical center charges.

## Results

Doctors diagnosed 304 patients (170 adults ages ≥16 years and 134 pediatric) with clinical malaria, prescribing antimalarial drugs to all. Giemsa thick smears were positive in 115/304 (38%). The typical patient cost for a Giemsa smear was 550 Naira (US$3.74 in 2009). For children, the cost of testing all, but treating only Giemsa positives was N888($6.04)/child; cost of empiric treatment of all who were clinically diagnosed was lower, N660($4.49)/child. For adults, the cost of testing all, but treating only Giemsa positives was N711($4.84)/adult for treatment option one (artesunate and sulfadoxine/ pyrimethamine) and N730($4.97)/adult for option two (artesunate and amodiaquine). This contrasts to lower costs of empiric treatment for both options one (N610=$4.14/adult) and two (N680=$4.63/adult).

## Conclusions

The most cost-effective approach to malarial management was empiric treatment, though many uninfected Nigerians will receive antimalarial drugs. If diagnostic costs declined, the cost-effectiveness calculus would change accordingly.

